# The Chancellor's Offer to the Doctors

**Published:** 1912-11-02

**Authors:** 


### The Hospital

The Workers' Newspaper of Administrative Medicine and Institutional Life, Administration, National Insurance and Health.

No. 1374, Vol. LIII. SATURDAY, NOVEMBER 2, 1912. PRICE ONE PENNY.

### THE CHANCELLOR'S OFFER TO THE DOCTORS.

Last week we published an outline of Mr. Lloyd George's new proposals to Parliament and to the medical profession concerning the remuneration to be offered to those members of the latter who accept service under the National Insurance Act. The British Medical Association, which represents so large a proportion of the doctors that for all practical purposes it may be regarded as their official mouthpiece, has not yet had time to come to any definite conclusion as to acceptance or rejection of these new terms. In offering a few comments upon Mr. George's proposals, we do not claim to speak for or to advise the profession, but merely to analyse the position from an independent standpoint as the institutional journal keenly interested in the whole of the issues involved in the vast scheme of National Insurance.

It is in the Chancellor's speech to the Advisory Committee that the new scheme is explained in detail; and that speech is one which repays close attention. It reveals a very clear comprehension by Mr. George of the difficulties of the profession, and of their reasonable objections to his original proposals. Indeed, we have seldom seen the case for the doctors better stated than in some passages of the speech. The Chancellor also made the astonishing admission that he has realised all along that his original proposals were inadequate, and that he has always contemplated concessions in the direction of greater liberality. The fact, of course, is that the concessions made, such as they are, have been wrung by *force majeure*, and that the Chancellor, having been beaten, is accepting defeat with as good grace as he can muster. But the calm admission that he has tried hard for months to sweat the doctors is one which is not likely to be forgotten.

The terms themselves are a very great advance on those previously offered. They fall short in many respects of the full demands of the British Medical Association, and some of the celebrated six points are flatly refused. Yet, in spite of this, the medical profession will do well, in our opinion, to think twice before it refuses them. Not that we attach any importance to Mr. George's thinly veiled threats of a National Medical Service; the doctors have only to stand firm and that bogey need not frighten them. But they have achieved much, and, in our opinion, the new terms deal not unjustly with them, so far as actual monetary remuneration goes. They will, of course, have to keep a very close eye on the details of the scheme when it comes to be put in black and white. The Chancellor is a past master of the art of window-dressing, and also of taking away with his right hand that which he gives with his left.

The new scheme offers seven shillings per insured person per annum to the doctor, exclusive of drugs, but inclusive of extras and mileage, and tuberculosis cases not under sanatorium treatment. If extras and mileage were struck out and provided for separately we think this offer would be a very fair one; as it is, the amount which will be absorbed by those two items is so vague (and so variable in different districts) that we regard their inclusion in the seven shillings flat rate as most unsatisfactory. Then there is one shilling and sixpence allotted to drugs. Whether this will be enough is very uncertain. The Chancellor says it will; German experience invites a contrary view. In towns this sum will go to the chemist; in remote districts, where the doctor himself will dispense, owing to the fact that no chemist exists, it will go to the doctor. Lastly, there is another sixpence, which is to go to the chemist if the one and six proves insufficient, but to the doctor otherwise. This is a very ingenious device. The fact that the chemist's one and sixpence cannot be raided by the doctor, no matter how he skimps his patients in the matter of drugs, will prevent under-drugging; while the fact that the extra sixpence will go to the doctor himself if he keeps the drug bill within the bounds of reason is a most potent inducement to reasonable economy. Still, further, there is an extra central fund to be drawn upon for drugs whenever unusual epidemics render the strain unduly heavy.

The Chancellor, after explaining these proposals, proceeded to impose certain conditions. First, the doctor is to provide free all certificates required to enable the patient to draw sick benefits. This condition we regard as reasonable, provided that the Chancellor is held to the exact words he used about it—that is, that such certificates are merely to certify the fact of ability or disability for work, and not to include the diagnosis or any report upon the facts whereon the diagnosis has been based. Next, the doctor is to keep simple records of the illnesses of, and attendances upon, his patients. Here, again, if the Chancellor means what he says about the "simplest character" of the records, we see no objection. Thirdly, there is to be some supervision of the services given by the doctor to ensure that they shall reach a proper standard of efficiency. This is a novel proposition, and one which seems to stick in the gizzard of many members of the profession. We do not see why it need; those who do their duty have nothing to fear from it. Rather in our opinion the doctors should bend their energies and their organisation to securing more definite and more generous arrangements over extras and mileage while accepting the proposed terms in other respects. As for the income limit and the other controversial demands, the best thing would be to drop them, making them a lever for securing the further concessions over extras, to which allusion has just been made.

